# Resveratrol Mimics Exercise-Induced Metabolic Stress to Suppress CIP2A and Epithelial–Mesenchymal Transition in 3D Renal Carcinoma Spheroids

**DOI:** 10.3390/biomedicines14030599

**Published:** 2026-03-08

**Authors:** Bang Sub Lee, Jong-Shik Kim, Wi-Young So

**Affiliations:** 1 Jeonbuk Sports Science Center, 62 Deulsapyeong-ro, Deokjin-gu, Jeonju-si 54894, Jeollabuk-do, Republic of Korea; lbs2427@jbsports.or.kr; 2 Department of Sports Science, Wonkwang University, Iksan-si 54538, Jeollabuk-do, Republic of Korea; 3 Department of Sports Medicine, College of Humanities, Korea National University of Transportation, Chungju-si 27469, Chungcheongbuk-do, Republic of Korea

**Keywords:** 3D culture, CIP2A, EMT inhibition, Exercise mimetic, Renal cell carcinoma, Resveratrol

## Abstract

**Background/Objectives:** We evaluated a 6-day repeated resveratrol exposure regimen in a three-dimensional (3D) culture model of human renal cell carcinoma (Caki-1) spheroids to examine phenotypic responses and changes in CIP2A abundance and epithelial–mesenchymal transition (EMT)-associated marker expression. **Methods:** Over 6 days, we assessed morphology and 2D cell viability and quantified CIP2A, fibronectin, and α-SMA by immunoblotting and immunofluorescence. **Results:** Resveratrol reduced 2D viability and increased cytoplasmic vacuoles, consistent with a stress-associated morphological response. In 3D spheroids, resveratrol treatment was associated with reduced CIP2A protein levels and decreased fibronectin and α-SMA, consistent with attenuation of a mesenchymal marker profile. **Conclusions:** These proof-of-concept data link 6-day resveratrol exposure to CIP2A reduction and decreased mesenchymal marker expression in a human 3D RCC spheroid system; however, PP2A activity and downstream signaling, AMPK/SIRT1 activation, and EMT-relevant functional assays were not assessed, and validation across additional RCC models will be required.

## 1. Introduction

Regular physical activity is associated with reduced cancer incidence and progression, including renal cell carcinoma (RCC), the most common malignancy of the adult kidney. Epidemiological studies consistently report improved outcomes among physically active individuals [1,2,3], and proposed mechanisms include modulation of cellular metabolism, oxidative stress responses, and oncogenic signaling pathways [4]. However, the molecular basis by which exercise-related cues influence tumor biology remains incompletely defined, in part because experimental systems that model exercise-like physiological stress in cancer are limited [5].

Pharmacological “exercise-mimetic” compounds have been used as tools to probe exercise-associated signaling in vitro [6]. Resveratrol has been reported to elicit exercise-mimetic metabolic signaling, frequently involving AMP-activated protein kinase (AMPK)/sirtuin 1 (SIRT1), and to modulate EMT-related programs in diverse models; however, whether these pathways mediate the observed effects in Caki-1 3D spheroids remains to be determined [7,8,9,10,11]. Resveratrol also exhibits anticancer activities in multiple preclinical models [12,13], yet how it relates to exercise-associated molecular features in RCC—particularly in tumor-mimetic 3D systems—remains underexplored.

A pathway of interest in RCC progression is the protein phosphatase 2A (PP2A) axis. The cancerous inhibitor of PP2A (CIP2A) is an oncoprotein that inhibits the tumor-suppressive phosphatase PP2A and has been linked to stabilization of c-Myc and pro-survival signaling [14,15,16]. CIP2A overexpression has been reported in RCC and is associated with adverse clinical outcomes and therapeutic resistance [17,18]. CIP2A has also been implicated in epithelial–mesenchymal transition (EMT)-associated programs through signaling networks that include c-Myc and PI3K/AKT pathways [17,19], suggesting that CIP2A may connect oncogenic signaling with mesenchymal marker regulation in RCC.

Model selection is critical for evaluating such responses. Conventional 2D monolayer cultures do not capture key architectural and microenvironmental features of solid tumors, whereas 3D spheroid and organoid systems better reflect spatial organization and diffusion constraints relevant to tumor biology [20,21,22]. These 3D platforms may therefore provide a more appropriate context to examine molecular responses to pharmacological stimuli intended to model exercise-related cues.

In this study, we used a human Caki-1 3D spheroid model to examine whether a 6-day repeated resveratrol exposure regimen is associated with changes in CIP2A abundance and EMT-associated marker expression in a tumor-mimetic 3D context. By focusing on the CIP2A/PP2A axis and EMT-related markers within a 3D RCC system, we aimed to provide proof-of-concept insight into resveratrol-responsive molecular features relevant to exercise-associated signaling hypotheses in renal carcinoma.

## 2. Materials and Methods

### 2.1. Materials

Resveratrol (purity ≥ 99%) was obtained from Sigma-Aldrich (St. Louis, MO, USA; catalog no. R5010) and dissolved in dimethyl sulfoxide (DMSO; Sigma-Aldrich) to prepare a 100 mM stock solution. Stock aliquots were stored at −20 °C and diluted in complete cell culture medium immediately before each experiment. The final DMSO concentration was consistently maintained below 0.1% in all experimental groups, including vehicle controls. Human RCC Caki-1 cells were purchased from the American Type Culture Collection (ATCC, Manassas, VA, USA; HTB-46) and cultured in Dulbecco’s Modified Eagle Medium (DMEM; Gibco, Thermo Fisher Scientific, Waltham, MA, USA), supplemented with 10% fetal bovine serum (FBS; Gibco), 100 U/mL penicillin, and 100 μg/mL streptomycin. Cells were incubated at 37 °C in a 5% CO_2_ humidified atmosphere. For conventional 2D culture, cells were seeded in standard tissue culture-treated plates (SPL Life Sciences, Seoul, Republic of Korea). For 3D culture, cells were seeded at a density of 1 × 10^6^ cells per well into ultra-low attachment six-well plates (Corning Inc., Corning, NY, USA; cat. no. 3471) and maintained for 6 d under non-adherent conditions to allow spontaneous spheroid formation. The following primary antibodies were used: anti-CIP2A (Santa Cruz Biotechnology, Dallas, TX, USA; sc-80659; RRID:AB_1121640), anti-fibronectin (Santa Cruz Biotechnology; sc-271098; RRID:AB_10608215), anti-α-smooth muscle actin (α-SMA; Abcam, Cambridge, UK; ab5694; RRID:AB_10608215), anti-E-cadherin (Abcam; ab76319; anti-rabbit), anti-N-cadherin (Abcam; ab76011; RRID: AB_1310479), and anti-β-actin (Santa Cruz Biotechnology; sc-47778; RRID:AB_626632). Alexa Fluor^®^ 488–conjugated goat anti-mouse IgG (H + L) cross-adsorbed secondary antibody (Invitrogen™, Thermo Fisher Scientific, Waltham, MA, USA; Cat. No. A-11001; RRID:AB_2534069) and Alexa Fluor^®^ 594–conjugated goat anti-rabbit IgG (H + L) cross-adsorbed secondary antibody (Invitrogen™, Thermo Fisher Scientific; Cat. No. A-11012; RRID:AB_2534079) were used for immunofluorescence staining. Hoechst 33342 nuclear stain (Thermo Fisher Scientific; Cat. No. H3570; RRID:AB_3675235) was used for nuclear counterstaining. Ethical approval was not required for this study as it utilized commercially available cell lines.

### 2.2. Cytotoxicity Assay and Dose Selection

To evaluate the concentration-dependent anticancer effects of resveratrol on human RCCs, Caki-1 cells were seeded into 96-well culture plates (SPL Life Sciences, Geumgang-ro, Korea) at a density of 3 × 10^3^ cells per well in 100 μL of complete DMEM. After 24 h of incubation, resveratrol was administered at various concentrations. Resveratrol was first dissolved in DMSO to create a 100 mM stock solution and subsequently diluted in culture medium to yield a maximum working concentration of 1 mM. A two-fold serial dilution was performed to generate a range of treatment concentrations from 1 mM to 0.0156 mM. DMSO content was maintained below 0.1% in all treatment and control groups. Cell viability was monitored at 24, 48, 72, and 96 h post-treatment using the WST-1 colorimetric assay (Roche Applied Science, Mannheim, Germany), according to the manufacturer’s instructions. At each time point, 10 μL of WST-1 reagent was added directly to each well, followed by incubation for 2 h at 37 °C. Absorbance was measured at 450 nm using a microplate reader (BioTek Instruments, Winooski, VT, USA), with background correction at 650 nm. All experimental conditions were performed in triplicate and repeated independently three times. The percentage of viable cells was calculated by normalizing absorbance values to those of untreated controls. Based on the resulting dose–response profiles and estimated IC_50_ values at each time point, non-cytotoxic concentrations of resveratrol (10 and 50 μM) were selected for further mechanistic evaluation using the 3D spheroid model.

### 2.3. Six-Day Repeated Exposure Protocol Using Caki-1 3D Spheroids

To model prolonged exposure to resveratrol under tumor-mimicking 3D conditions, Caki-1 RCCs were seeded into ultra-low-attachment six-well plates (Corning Inc.) at a density of 3 × 10^5^ cells per well. Cells were cultured in DMEM supplemented with 10% FBS, 1% penicillin–streptomycin, and 1% L-glutamine. To support extracellular matrix (ECM)-associated 3D organization during spheroid formation, Matrigel^®^ (Corning; [Cat. No. A4000046902]) was added at 1 mL per 3 wells at the time of seeding and gently mixed. Under non-adherent conditions, spheroids formed spontaneously over a 6 d period. Resveratrol treatment (10 or 50 μM) commenced on day 0, and the drug-containing medium was replaced on day 3 to maintain consistent exposure throughout the culture period. The vehicle control group received DMSO at a final concentration of <0.1%, following the same treatment schedule. At the end of the treatment period (day 6), spheroids were harvested for downstream analyses. Spheroid size at treatment initiation (day 0) and at harvest (day 6) was quantified from bright-field images by measuring the 2D projected area and calculating the area-equivalent diameter using ImageJ (version 1.53, NIH, Bethesda, MD, USA); objects intersecting the image boundary were excluded. Representative images and size quantification are provided in Supplementary Figure S2. Immunofluorescence staining was conducted to visualize the expression patterns of CIP2A, fibronectin, and α-SMA, whereas Western blotting was performed to validate protein expression changes. This experimental approach was designed to recapitulate the metabolic and morphological adaptations observed under exercise-mimetic conditions, utilizing a 3D tumor model that mimics the in vivo architecture and cellular interactions of RCC. Through this system, we sought to determine whether resveratrol modulates oncogenic signaling and EMT-related processes within a physiologically relevant tumor-like microenvironment.

### 2.4. Immunofluorescence Staining for CIP2A and Epithelial–Mesenchymal Marker Analysis

To evaluate the spatial expression of CIP2A and key markers associated with EMT, Caki-1 spheroids were subjected to immunofluorescence staining and analyzed using a high-content imaging system (Operetta CLS; PerkinElmer, Waltham, MA, USA). After 6 d of resveratrol exposure, spheroids were collected and fixed with 4% paraformaldehyde for 20 min at room temperature. Following phosphate-buffered saline (PBS) washes, samples were permeabilized using 0.1% Triton X-100 for 10 min and subsequently blocked with 2% bovine serum albumin in PBS for 1 h to minimize non-specific antibody binding. Spheroids were incubated overnight at 4 °C with primary antibodies targeting CIP2A (1:100), fibronectin (1:100), and α-SMA (1:200). Subsequently, samples were washed and incubated with Alexa Fluor–conjugated secondary antibodies (1:1000 dilution) for 1 h at room temperature in the dark. Nuclear staining was performed with Hoechst 33342 (2 µg/mL) for 10 min. Stained spheroids were transferred to CellCarrier-384 ultra-clear plates (PerkinElmer) for imaging. Fluorescence images were acquired as confocal z-stacks under uniform exposure settings across all samples, enabling high-resolution visualization of marker distribution. Quantitative image analysis, including intensity measurement and localization mapping, was performed using Harmony High Content Analysis Software (version 4.9, PerkinElmer, Waltham, MA, USA). This approach provided robust and reproducible insight into how resveratrol influences CIP2A and EMT-related protein expression within the intact 3D architecture of renal carcinoma spheroids. Fluorescence intensity and marker distribution were quantified using ImageJ (NIH, Bethesda, MD, USA).

### 2.5. Western Blot Analysis

To investigate changes in CIP2A and EMT-associated protein levels following resveratrol exposure, Western blot analysis was conducted using protein lysates from Caki-1 spheroids cultured under 3D conditions. After 6 d of treatment, spheroids were carefully collected and lysed in RIPA buffer (Thermo Fisher Scientific, Waltham, MA, USA) supplemented with protease and phosphatase inhibitor cocktails (Roche Diagnostics, Indianapolis, IN, USA). Lysis was performed on ice for 30 min to preserve protein integrity. Lysates were centrifuged at 14,000× *g* for 15 min at 4 °C, and the resulting supernatants were harvested. Protein concentrations were measured using the BCA Protein Assay Kit (Pierce Biotechnology, Rockford, IL, USA) according to the manufacturer’s instructions. Due to limited protein yield from 3D spheroids, samples from three independent culture runs were pooled per condition prior to lysis to obtain sufficient material for immunoblotting; accordingly, immunoblots were generated from pooled samples run once per target/membrane and are presented as confirmatory results. Equal amounts of total protein (20–30 µg per lane) were mixed with Laemmli sample buffer, boiled at 95 °C for 5 min, and resolved by SDS–polyacrylamide gel electrophoresis. Proteins were transferred to PVDF membranes (Millipore, Billerica, MA, USA) using a conventional wet transfer system. Membranes were blocked for 1 h in 5% non-fat dry milk prepared in Tris-buffered saline with 0.1% Tween-20 to prevent non-specific antibody binding. Primary antibodies against CIP2A (1:500), fibronectin (1:500), α-SMA (1:1000), and β-actin (1:1000, used as a loading control) were applied and incubated overnight at 4 °C. After washing, membranes were incubated for 1 h at room temperature with horseradish peroxidase-conjugated secondary antibodies (1:2000 dilution). Immunoreactive bands were visualized using enhanced chemiluminescence reagents (Thermo Scientific, Waltham, MA, USA) and detected using a ChemiDoc™ MP imaging system (Bio-Rad Laboratories, Hercules, CA, USA). Band intensities were quantified using Image Lab software (Version 6.1; Bio-Rad Laboratories, Inc., Hercules, CA, USA), and relative levels were normalized to β-actin. This method allowed for accurate quantification of resveratrol-induced protein expression alterations in a 3D renal cancer model.

### 2.6. Statistical Analysis

Statistical analyses were performed using GraphPad Prism version 9.5.1 (GraphPad Software, San Diego, CA, USA). Quantitative data are presented as mean ± standard deviation (SD), unless otherwise stated. For immunofluorescence quantification, individual spheroids were treated as the biological unit (*n* = number of spheroids analyzed per condition; n = 5 spheroids per condition). When multiple images (fields) were acquired from a single spheroid, values were averaged to yield one value per spheroid for statistical analysis. Differences among groups were evaluated using one-way analysis of variance (ANOVA) followed by Dunnett’s multiple comparisons test for comparisons versus the vehicle control group. A *p*-value < 0.05 was considered statistically significant. Because Western blot samples were generated from pooled spheroid lysates due to limited protein yield, independent replicate blots (biological n) could not be defined; therefore, Western blots are presented as confirmatory results and were not used for statistical inference based on densitometry.

## 3. Results

### 3.1. Resveratrol Reduces Cell Viability and Induces Vacuole Formation in Caki-1 RCCs

**Figure 1:**
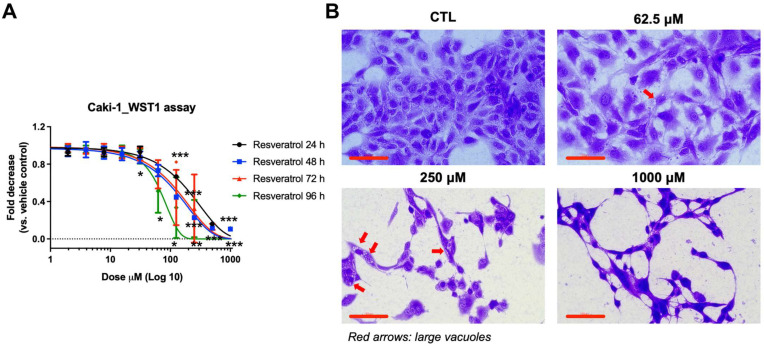
Resveratrol reduces cell viability and induces early vacuole formation in Caki-1 cells. (**A**) Cell viability of Caki-1 cells was assessed using the WST-1 assay after 24, 48, 72, and 96 h exposure to resveratrol at various concentrations under 2D culture conditions. Statistical significance was determined for each time point relative to the corresponding vehicle control (CTL). * *p* < 0.05, ** *p* < 0.01, *** *p* < 0.001 (vs. CTL). Resveratrol treatment resulted in a dose-dependent reduction in viability. Data are presented as mean ± SD (n = 12, 3 different tests). (**B**) Representative crystal violet-stained image of Caki-1 cells after 96 h treatment. Control cells exhibit normal epithelial morphology with tight cell-to-cell junctions, while resveratrol-treated cells show early cytoplasmic vacuole formation (red arrow). Scale bar = 100 µm.

To investigate the cytotoxic effects of resveratrol in human RCCs, Caki-1 cells were cultured under standard 2D conditions and treated with varying concentrations of resveratrol for 24, 48, 72, and 96 h. Cell viability was assessed using the WST-1 assay. As illustrated in Figure 1A, resveratrol induced a concentration-dependent decrease in viability at all time points, with pronounced reductions following prolonged exposure. At concentrations of ≥25 µM, viability dropped significantly 48 h post-treatment, suggesting that even moderate doses exert anti-proliferative effects over time. Notably, this reduction was gradual rather than abrupt, indicating a cumulative cellular stress response rather than acute toxicity. To further characterize resveratrol-induced morphological changes, cells were stained with crystal violet following 96 h exposure. Control cells exhibited normal epithelial morphology with round, closely packed cells and intact cell–cell junctions (Figure 1B). Conversely, resveratrol-treated cells showed dispersed morphology, cytoplasmic shrinkage, and the formation of large cytoplasmic vacuoles (red arrows). These vacuoles likely represent early indicators of metabolic stress and cellular remodeling, possibly associated with autophagic or vacuolar death pathways. These findings indicate that resveratrol, even at sub-lethal doses, can initiate morphological changes reflective of stress adaptation or cell death.

### 3.2. Development of a 3D Spheroid Model for 6-Day Repeated, Low-Dose Resveratrol Exposure

**Figure 2:**
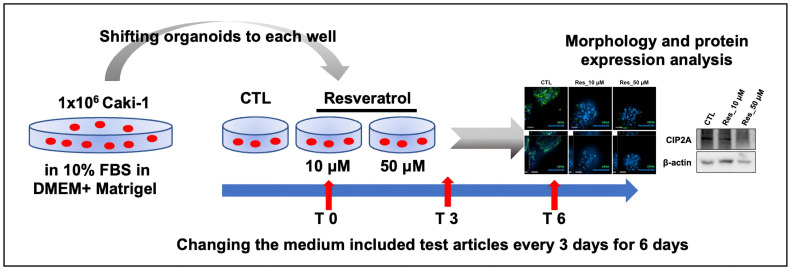
Schematic diagram of a 6-day repeated low-dose resveratrol exposure in 3D-cultured Caki-1 spheroids. Caki-1 cells were seeded in ultra-low attachment 6-well plates and allowed to form 3D spheroids over 6 d. Starting on day 0, resveratrol was administered at low concentrations and refreshed every 3 d over a total of 6 d. Spheroids were harvested on day 6 for immunofluorescence staining and Western blot analyses to assess protein expression levels and morphological changes. This model was used to apply a repeated resveratrol exposure regimen in a tumor-mimetic 3D environment.

Given the limitations of short-term 2D culture systems in recapitulating in vivo-like drug responses, we established a 3D spheroid model using Caki-1 cells. As illustrated in Figure 2, cells were seeded in ultra-low-attachment plates, which promoted spheroid formation over a 6 d period. Starting on day 0, resveratrol was administered at 10 or 50 µM and replenished every 3 days to maintain a 6-day repeated exposure regimen. Spheroids were harvested on day 6 for analysis. This experimental setup was specifically designed to model short-term metabolic stress similar to the physiological adaptations induced by exercise. We aimed to assess whether resveratrol, recognized for its exercise-mimetic molecular effects via SIRT1 and AMPK activation, exerts similar modulatory effects on RCCs under sustained exposure. The 3D culture format more accurately recapitulates tumor-like architecture and microenvironmental conditions, including drug diffusion gradients and stress responses, offering a more relevant platform for evaluating subtle phenotypic changes induced by bioactive compounds.

### 3.3. Resveratrol Downregulates CIP2A Expression in 3D-Cultured Caki-1 Spheroids

**Figure 3:**
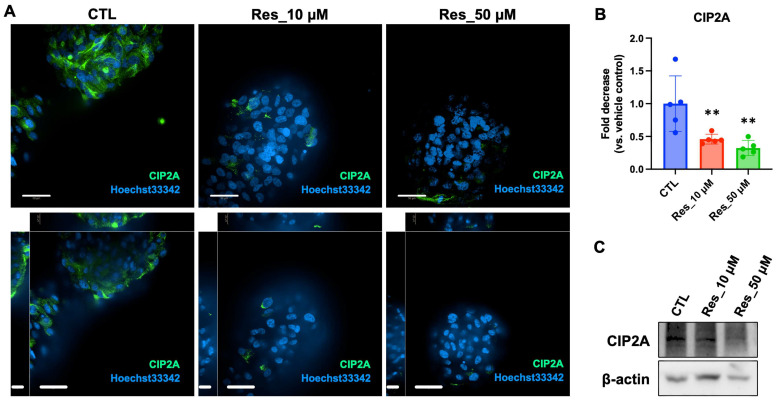
A 6-day repeated resveratrol treatment suppresses CIP2A expression in 3D-cultured Caki-1 renal carcinoma spheroids. (**A**) Representative immunofluorescence images of 3D Caki-1 spheroids following a 6-day resveratrol exposure regimen (CTL, 0 μM; 10 μM; 50 μM). CIP2A (red) and nuclei (Hoechst 33342, blue) are shown; merged images are presented (Scale bars: short = 20 μm, long = 50 μm). (**B**) Quantification of CIP2A fluorescence intensity using ImageJ. Individual spheroids were treated as the biological unit (n = 5 spheroids per condition). Data are presented as mean ± SD. Statistical analysis was performed using one-way ANOVA followed by Dunnett’s multiple comparisons test versus CTL (** *p*< 0.01 (vs. CTL)). (**C**) Representative Western blot showing CIP2A protein levels in 3D spheroids, with β-actin as a loading control. Due to limited protein yield, spheroids from three independent culture runs were pooled per condition prior to lysis; immunoblotting was performed on a limited number of pooled blots (two gel runs/membranes) and is presented as confirmatory evidence. Uncropped blots with molecular weight markers are provided in Supplementary Figure S1.

CIP2A is an oncoprotein that promotes tumor progression by suppressing the tumor suppressor phosphatase PP2A. To investigate whether resveratrol modulates CIP2A expression under physiologically relevant conditions, we cultured human renal carcinoma Caki-1 cells as 3D spheroids, which more accurately recapitulate in vivo tumor architecture than 2D monolayers. Caki-1 spheroids were treated with resveratrol at concentrations of 0 μM (CTL), 10 μM, and 50 μM for 6 d. As shown in Figure 3A, CIP2A protein (green) was prominently expressed in untreated spheroids, exhibiting diffuse cytoplasmic localization. Conversely, resveratrol treatment induced a concentration-dependent reduction in CIP2A fluorescence intensity, with the 50 μM group exhibiting a marked decrease. Nuclear morphology, visualized using Hoechst 33342 (blue), remained intact across all groups, suggesting that CIP2A downregulation precedes detectable nuclear damage.

Quantitative image analysis using ImageJ (Figure 3B) confirmed a statistically significant decrease in CIP2A signal intensity in resveratrol-treated spheroids compared with the CTL group. Western blot analysis (Figure 3C) further validated these findings, with β-actin serving as a loading control. Densitometric analysis showed that band intensities normalized to β-actin were markedly lower in the resveratrol-treated groups. Collectively, these findings indicate that resveratrol effectively suppresses CIP2A expression in 3D-cultured renal carcinoma spheroids, which may contribute to the attenuation of oncogenic signaling and EMT in this model.

### 3.4. Resveratrol Suppresses Mesenchymal Marker Expression in 3D-Cultured Caki-1 Spheroids

**Figure 4:**
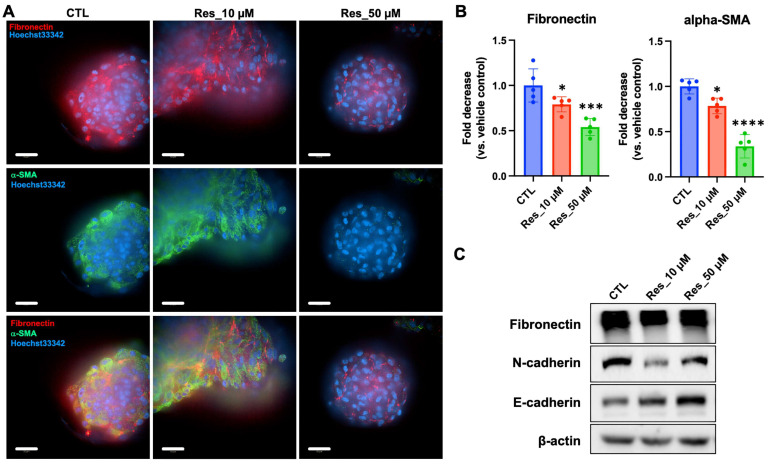
A 6-day repeated resveratrol exposure regimen reduces mesenchymal marker expression in 3D Caki-1 renal carcinoma spheroids. (**A**) Representative immunofluorescence images of 3D Caki-1 spheroids following a 6-day repeated resveratrol exposure regimen (CTL, 0 μM; 10 μM; 50 μM). Spheroids were stained for fibronectin (red) and α-SMA (green), with nuclei counterstained with Hoechst 33342 (blue). Merged images are shown. (Scale bar: 50 μm). (**B**) Quantification of fibronectin and α-SMA fluorescence intensities using ImageJ. Individual spheroids were treated as the biological unit (n = 5 spheroids per condition). Data are presented as mean ± SD. Statistical analysis was performed using one-way ANOVA followed by Dunnett’s multiple comparisons test versus CTL (* *p* < 0.05, *** *p* < 0.001, **** *p* < 0.0001 (vs. CTL)). (**C**) Representative Western blots of fibronectin, N-cadherin, and E-cadherin in 3D spheroids, with β-actin as a loading control. Due to limited protein yield, spheroids from three independent culture runs were pooled per condition prior to lysis; immunoblotting was performed on a limited number of pooled blots (two gel runs/membranes) and is presented as confirmatory evidence. Uncropped blots with molecular weight markers are provided in Supplementary Figure S1. CTL, vehicle control.

To determine whether resveratrol modulates EMT-related phenotypes in renal carcinoma, we evaluated the expression of representative mesenchymal markers in 3D-cultured Caki-1 spheroids after 6 days of treatment. Immunofluorescence staining revealed that vehicle-treated control spheroids (CTL) exhibited strong fibronectin (red) and α-SMA (green) signals, which were largely enriched along the spheroid periphery (Figure 4A). In contrast, resveratrol treatment (10 or 50 μM) reduced the intensity of both markers in a concentration-dependent manner, with the 50 μM group showing the most pronounced attenuation. Consistent with these observations, merged images showed an overall decrease in peripheral fibronectin/α-SMA signals in resveratrol-treated spheroids, suggesting suppression of EMT-associated extracellular matrix and contractile features. Quantification of fluorescence intensity using ImageJ confirmed significant reductions in fibronectin and α-SMA levels relative to CTL (Figure 4B). Specifically, fibronectin was significantly decreased at 10 μM and further reduced at 50 μM, while α-SMA showed a similar dose-responsive decline (Figure 4B). To further assess EMT-related protein changes at the bulk level, we performed Western blot analysis of fibronectin and cadherin markers (Figure 4C). Resveratrol exposure decreased fibronectin expression and was accompanied by alterations in cadherin profiles, with reduced N-cadherin (notably at 10 μM) and increased E-cadherin at 50 μM (Figure 4C). β-actin served as a loading control, and band intensities were normalized accordingly. Collectively, these data indicate that resveratrol suppresses mesenchymal marker expression and shifts EMT-associated protein patterns toward a more epithelial-like state in Caki-1 spheroids, supporting its potential to modulate EMT-related cellular phenotypes within a physiologically relevant 3D tumor microenvironment.

## 4. Discussion

Physical activity is associated with a reduced risk of cancer incidence and progression, including RCC [23]. Epidemiological data suggest that regular exercise contributes to improved metabolic homeostasis, reduced systemic inflammation, and modulation of signaling pathways involved in tumor development and progression [24,25]. However, despite these well-established correlations, direct experimental evidence elucidating how exercise-like stimuli affect human cancer cells at the molecular level—particularly within tumor-mimetic 3D environments—remains limited.

In this study, we addressed this gap by employing a 3D culture model of human renal carcinoma (Caki-1) spheroids and using resveratrol as a pharmacological stimulus that has been reported to engage exercise-related metabolic regulators such as SIRT1 and AMPK [26,27]. These pathways have been implicated in exercise-associated benefits, including regulation of energy balance, mitochondrial homeostasis, and cellular stress responses [28]. Accordingly, resveratrol has been discussed in the literature as a candidate exercise-mimetic compound [29,30,31]. However, because we did not directly assess AMPK/SIRT1 activation or other metabolic stress markers in our model, we use the term “exercise-mimetic” in a conservative, literature-contextual sense and interpret our findings primarily at the level of observed phenotypic and molecular responses in 2D and 3D cultures. Our results demonstrate that resveratrol induces a dose-dependent reduction in Caki-1 cell viability under 2D culture conditions and promotes cytoplasmic vacuole formation, which we interpret as a stress-associated morphological change. The underlying pathways were not assessed in this study, and interpretation will require targeted validation using autophagy/apoptosis and stress signaling markers (e.g., LC3, p62, cleaved caspase-3, PARP, and AMPK/mTOR-related readouts) in future work. Because viability was assessed only in Caki-1 cells, the observed reduction should not be interpreted as tumor-selective cytotoxicity, and comparison with non-cancer renal epithelial cells (e.g., HK-2 or primary renal epithelial cells) will be required in future work to establish selectivity. Although these observations provide initial evidence of the cytostatic activity of resveratrol, the most compelling findings were obtained using the 3D spheroid model, which more closely recapitulates the spatial organization, diffusion gradients, and cell–cell interactions of in vivo tumors.

In this 3D context, resveratrol reduced the protein abundance of CIP2A (cancerous inhibitor of PP2A), an oncoprotein reported to inhibit the tumor suppressor phosphatase PP2A and stabilize c-Myc [32]. Given that CIP2A has been linked to oncogenic processes and poor prognosis in several cancers, including RCC [18], the observed reduction in CIP2A may be biologically relevant. However, PP2A enzymatic activity and downstream signaling readouts (e.g., c-Myc abundance and AKT/ERK phosphorylation) were not measured in this study; therefore, we cannot determine whether CIP2A reduction was accompanied by PP2A reactivation or pathway-level modulation. Notably, CIP2A reduction co-occurred with decreased expression of mesenchymal markers, including fibronectin and α-SMA, as assessed by immunofluorescence and Western blot analyses. EMT is a dynamic process through which epithelial tumor cells acquire mesenchymal features and is associated with metastasis, drug resistance, and immune evasion [33]. While these marker-level changes are consistent with attenuation of an EMT-associated molecular phenotype under resveratrol treatment in this 3D model, functional assays relevant to EMT were not performed (e.g., migration, invasion, wound-healing, or spheroid dispersal/outgrowth). Therefore, we do not infer changes in motility or invasive behavior from the current dataset, and a causal relationship between CIP2A downregulation and EMT-marker changes cannot be concluded. Taken together, our findings support a working hypothesis that resveratrol may influence EMT-associated signaling through a CIP2A–PP2A-related mechanism. This proposed CIP2A–PP2A–EMT axis warrants future validation by directly measuring PP2A activity and downstream signaling intermediates (including c-Myc and AKT/ERK phosphorylation) and by applying CIP2A loss-of-function and rescue experiments [34].

A strength of this study is the use of a 3D renal carcinoma organoid model, which enabled the observation of phenotypic and molecular responses in a context that more closely mirrors in vivo tumor physiology. Compared with traditional 2D cultures, 3D spheroids provide a more accurate platform to study drug responses, cellular heterogeneity, and microenvironmental interactions. By applying resveratrol in this setting, we show that resveratrol treatment is associated with reduced CIP2A abundance and attenuation of mesenchymal marker expression in a human 3D Caki-1 RCC spheroid model.

Our findings have several implications. First, our findings support resveratrol as a candidate compound for further evaluation in RCC models. However, the nominal concentrations used here (10–50 µM) may exceed systemically achievable levels in vivo because resveratrol has limited bioavailability and undergoes rapid metabolism. Future studies should incorporate exposure-aligned designs (e.g., lower concentrations, repeated low-dose regimens, and/or bioavailability-enhancing formulations) to strengthen translational interpretation. Translational considerations. The translational interpretation of resveratrol in RCC is constrained by poor oral bioavailability and rapid metabolism, which can limit sustained systemic exposure. Accordingly, our findings should be interpreted as proof-of-concept observations in an in vitro 3D spheroid system rather than evidence of clinically achievable exposure or therapeutic efficacy. Future work should consider exposure-aligned dosing strategies and bioavailability-enhancing delivery approaches, together with validation in additional RCC models, including patient-derived systems. Second, they highlight the utility of candidate exercise-mimetic compounds as experimental tools for dissecting exercise-related tumor biology in human organoid systems. Finally, they underscore the feasibility of using cancer organoids to model complex physiological stresses—such as those induced by physical activity—within a controlled laboratory setting. Notably, matched molecular profiling of CIP2A and EMT-associated markers in 2D cultures was not performed in this study and will be addressed in future work to enable direct 2D–3D comparisons. Future studies could leverage transcriptomic and phosphoproteomic profiling to identify downstream pathways altered by CIP2A inhibition, alongside functional assays to assess changes in migration, invasion, and resistance phenotypes. Determining whether the EMT-associated marker changes observed here extend to other RCC subtypes or are limited to Caki-1 cells would provide insights into its broader applicability. Moreover, comparing the effects of resveratrol with exercise-conditioned serum or mechanical stress models could validate its role as a surrogate for physical activity in cancer modulation.

Despite these implications, several limitations should be considered when interpreting the present findings. A key limitation of this study is that the experiments were performed using a single human RCC cell line (Caki-1) in 2D culture and 3D spheroid formats. RCC is molecularly and clinically heterogeneous, and responses to resveratrol may differ across subtypes (e.g., clear cell, papillary, and chromophobe RCC) and across patient-derived tumors. In addition, metabolic stress pathway activation was not directly evaluated (e.g., AMPK phosphorylation/activation, SIRT1 activity, mitochondrial function, ROS, or ATP), which limits mechanistic interpretation of resveratrol as an exercise-mimetic stimulus in this model. Relatedly, because AMPK/SIRT1 activation and downstream CIP2A–PP2A signaling events (e.g., PP2A activity, c-Myc stability, and AKT/ERK phosphorylation) were not measured, mechanistic causality linking metabolic stress to CIP2A suppression and EMT modulation cannot be concluded. Furthermore, functional endpoints relevant to EMT were not assessed (e.g., migration, invasion, wound-healing, or spheroid dispersal/outgrowth assays), limiting interpretation of the marker changes in terms of motility or invasive behavior. We also did not evaluate molecular markers required to interpret vacuole formation and stress responses (e.g., LC3/p62 for autophagy, cleaved caspase-3/PARP for apoptosis, and AMPK/mTOR pathway readouts), which limits conclusions regarding the underlying stress mechanism. Therefore, the present findings should be interpreted as proof-of-concept observations within a Caki-1 spheroid context. Future work will validate reproducibility across multiple RCC cell lines representing distinct subtypes and, where feasible, in patient-derived organoids to assess subtype robustness and translational relevance. In parallel, we will test the proposed signaling framework by quantifying p-AMPK/p-ACC and SIRT1 activity, and by assessing PP2A activity together with canonical downstream substrates following resveratrol exposure.

Our study provides evidence that resveratrol treatment is associated with reduced CIP2A abundance and attenuation of mesenchymal marker expression in a human 3D Caki-1 RCC spheroid model; however, pathway-level validation and cross-model replication will be required to establish mechanistic and subtype generalizability. This 3D platform may be useful for exploring how exercise-related molecular cues and their pharmacological mimetics modulate tumor-associated phenotypes in vitro, and it provides a foundation for future studies evaluating translational relevance across diverse RCC models, including additional cell lines and patient-derived organoids.

## 5. Conclusions

In this study, we used a human Caki-1-derived 3D spheroid model to examine responses to a 6-day repeated resveratrol exposure regimen. Resveratrol treatment was associated with reduced CIP2A protein abundance and decreased mesenchymal marker expression (fibronectin and α-SMA) in 3D spheroids, accompanied by cytoplasmic vacuolization. These findings provide proof-of-concept evidence of resveratrol-responsive molecular and phenotypic changes in a tumor-mimetic 3D RCC context. However, pathway-level validation (e.g., PP2A activity and downstream signaling), metabolic stress marker assessment (e.g., AMPK/SIRT1-related readouts), EMT-relevant functional assays, and cross-model replication will be required to establish mechanistic interpretation and generalizability. Overall, our 3D platform offers a useful framework for future studies investigating exercise-related molecular cues and their pharmacological surrogates in RCC models.

## Data Availability

The data supporting the findings of this study are available from the corresponding author upon request.

## Supplementary Materials

The following supporting information can be downloaded at: https://www.mdpi.com/article/10.3390/biomedicines14030599/s1, Figure S1: Uncropped western blots for CIP2A and EMT-associated proteins in 3D-cultured Caki-1 spheroids after a 6-day repeated resveratrol exposure regimen; Figure S2: Representative bright-field images of Caki-1 spheroids at Day 0 and Day 6 used for size analysis.

## Abbreviations

The following abbreviations are used in this manuscript:

3D	Three-dimensional
α-SMA	Alpha smooth muscle actin
AMPK	AMP-activated protein kinase
CIP2A	Cancerous inhibitor of protein phosphatase 2A
DMSO	Dimethyl sulfoxide
DMEM	Dulbecco’s Modified Eagle Medium
EMT	Epithelial–mesenchymal transition
FBS	Fetal bovine serum
PBS	Phosphate-buffered saline
RCC	Renal cell carcinoma
Res	Resveratrol
ROS	Reactive oxygen species
WB	Western blot
