# Supplementary material for: Resveratrol Mimics Exercise-Induced Metabolic Stress to Suppress CIP2A and Epithelial–Mesenchymal Transition in 3D Renal Carcinoma Spheroids
Source: Biomedicines. 2026 Mar 8;14(3):599. doi: 10.3390/biomedicines14030599 (PMC13024014; doi:10.3390/biomedicines14030599)
Supplement: Supplementary file 1 [file biomedicines-14-00599-s001.zip › biomedicines-4157987-supplementary.pdf]

Supplementary Materials

for

Resveratrol mimics exercise-induced metabolic stress to sup-press CIP2A and epithelial–mesenchymal transition in 3D renal carcinoma spheroids

Bang Sub Lee 1, Jong-Shik Kim 2,*, and Wi-Young So 3,*

***Corresponding authors:** judozang@wku.ac.kr (J.-S.K.); wowso@ut.ac.kr (W.-Y.S.); Tel.: +82-63-850-6203 (J.-S.K.); +82-43-841-5991 (W.-Y.S.); Fax: +82-63-850-6095 (J.-S.K.); +82-43-841-5990 (W.-Y.S.)

This file contains Supplementary Figure S1-Figure S2.

Supplementary Figure S1 Uncropped western blots for CIP2A and EMT-associated proteins in 3D-cultured Caki-1 spheroids after a 6-day repeated resveratrol exposure regimen

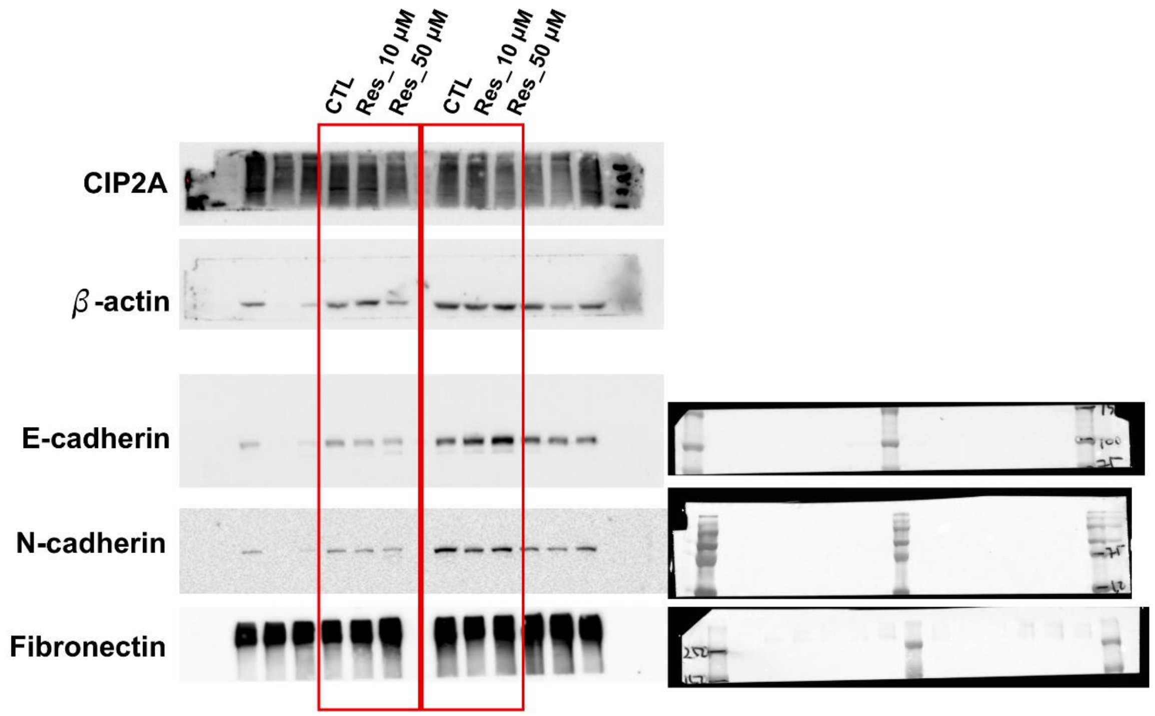

Figure S1. Uncropped immunoblots corresponding to the western blot panels shown in the main figures. 3D Caki-1 spheroids were treated with vehicle control (CTL) or resveratrol (Res; 10 or 50 μM) for 6 days, with resveratrol refreshed every 3 days. Due to limited protein yield from spheroids, lysates were prepared by pooling spheroids collected from three independent culture runs per condition to obtain sufficient material for gel loading; thus, the immunoblots are presented as confirmatory readouts. Membranes were probed for CIP2A and EMT-associated proteins (E-cadherin, N-cadherin, and fibronectin), with β-actin used as a loading control. Molecular weight markers are shown. Red boxes indicate the cropped regions used for the corresponding western blot panels in the main figures.

Supplementary Figure S2 Representative bright-field images of Caki-1 spheroids at Day 0 and Day 6 used for size analysis

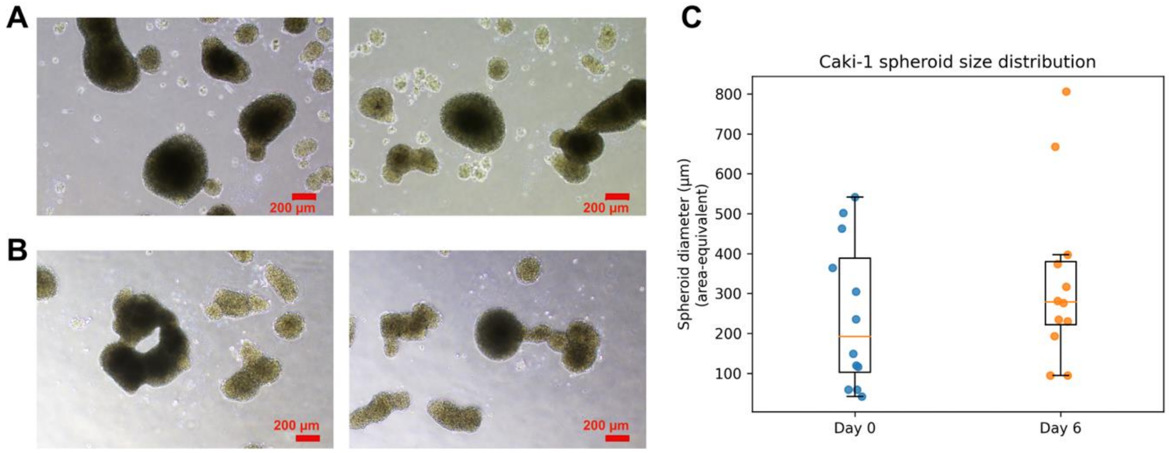

Figure S2. (A) Representative bright-field images of 3D-cultured Caki-1 spheroids at Day 0 (treatment initiation). (B) Representative bright-field images of Caki-1 spheroids at Day 6 (harvest). Scale bars: 200 μm. (C) Spheroid size distribution quantified from bright-field images. Spheroid diameter was calculated as the area-equivalent diameter from the 2D projected area (ImageJ-based analysis); objects intersecting the image boundary were excluded. Data were obtained from two representative fields per time point (Day 0: n = 12 spheroids; Day 6: n = 12 spheroids). Day 0: 246 ± 184 μm (median 192 μm; range 42–541 μm). Day 6: 330 ± 213 μm (median 278 μm; range 95–806 μm).
